# Maternal and Newborn-care Practices during Pregnancy, Childbirth, and the Postnatal Period: A Comparison in Three Rural Districts in Bangladesh

**Published:** 2006-12

**Authors:** S. Barnett, K. Azad, S. Barua, M. Mridha, M. Abrar, A. Rego, A. Khan, D. Flatman, A. Costello

**Affiliations:** ^1^ UCL Centre for International Health and Development, Institute of Child Health, University College London, 30 Guilford Street, London WC1N 1EH, UK; ^2^ Perinatal Care Project, Diabetic Association of Bangladesh, BIRDEM, Ibrahim Memorial Diabetic Centre, Shahbagh, Dhaka, Bangladesh; ^3^ Women and Children First, 30 Guilford Street, London WC1N 1EH

**Keywords:** Pregnancy, Safe motherhood, Newborn care, Healthcare-seeking behaviour, Cross-sectional studies, Retrospective studies, Baseline surveys, Bangladesh

## Abstract

The aim of this study was to examine the prevalence of maternal and newborn-care practices among women reporting a birth in the previous year in three districts in different divisions of Bangladesh. In 2003, 6,785 women, who had delivered a newborn infant in the previous year, across three districts in Bangladesh, were interviewed. Overall, less than half of the women received any antenatal care, and 11% received a minimum of four check-ups. Only 18% took iron tablets for at least four months during pregnancy. Over 90% of the 6,785 deliveries took place at home, and only 11% were attended either by a doctor or by a nurse. The mothers reported three key hygienic practices in 54% of deliveries: attendants washing their hands with soap and boiling cord-tie and blade for cutting the cord. Forty-four percent of the 6,785 infants were bathed immediately after delivery, and 42% were given colostrum as their first food. The results suggest that maternal and newborn-care remains a cause of concern in rural Bangladesh. Short-term policies to promote healthy behaviour in the home are needed, in addition to the long-term goal of skilled birth attendance.

## INTRODUCTION

Bangladesh has shown impressive improvements in health over the past 30 years. Between 1975 and 2004, the total fertility rate fell by more than 50% from 6.3 to 3.0 (1). The rate of mortality among children aged less than five years was estimated to be 180 per 1,000 livebirths in the 1979–1983 period (2) but had fallen to 88 per 1,000 livebirths in the 1999–2003 period (1). The rate of infant mortality fell from 117 (1979–1983) (2) to 65 per 1,000 livebirths (1999–2003) (1), and the rate of neonatal mortality fell from 78 (1979–1983) (2) to 41 per 1,000 livebirths (1999–2003) (1). Furthermore, Bangladesh is unusual among developing countries because, despite the very low level of skilled attendance at delivery (3) over the past decade, the rate of maternal mortality has fallen steadily from 470 in 1991 to 310 per 100,000 livebirths in 2001 (4). On the other hand, pregnancy-related complications are still one of the leading causes of death and disability among women of childbearing age in Bangladesh (5).

The Millennium Development Goal 4 calls for a reduction of two-thirds in mortality among children aged less than five years between 1990 and 2015. In Bangladesh, based on the aforementioned mortality rate of 88 per 1,000 livebirths and a neonatal mortality rate of 41 per 1,000 livebirths (1), 47% of deaths in children aged less than five years occur in the first month, and a reduction in neonatal mortality is, therefore, crucial to achieving this goal. The factors associated with reduced rates of neonatal mortality include antenatal care, clean delivery practices, prevention of hypothermia, early breastfeeding, and early care-seeking behaviour (6). The study examined the prevalence of these practices among women reporting a birth in the previous year in three districts in different divisions of Bangladesh. Qualitative anthropological papers help further understand the reasons behind these practices (7–9).

## MATERIALS AND METHODS

During January-March 2003, a cross-sectional survey was conducted to collect detailed retrospective information about pregnancy, childbirth, and the postnatal period in rural Bangladesh. Women were asked about their most recent pregnancy and, to limit recall-bias, only women who had delivered a baby within the last one year and lived in the study area were selected for interview. Background socioeconomic and demographic information about mothers and their households and their pregnancy history was also collected. The survey was conducted in Bogra, Faridpur, and Moulvibazar which were selected using purposive sampling from three different divisions. The study catchment area comprised two chosen upazilas (sub-districts) from each district, from which three unions were randomly selected from each upazila. The study area covered 18 unions, with a population of 480,425.

In each district, 12 interviewers were recruited and monitored by one field supervisor. The set of questionnaire was piloted outside the study area in November 2002 in six randomly-selected unions—two in each district. In total, 120 questionnaire—20 in each union—were pre-tested by the interviewers. The number of mothers to be interviewed in each union was weighted according to the total population of the union based on the 1991 Census. Each union is divided into nine wards. Since ward-level population data were not available, the number of women interviewed in each union was divided equally between the wards. The central point of each ward was identified, and the interviewers spun a bottle to randomly select a direction. Subsequently, the interviewers visited every household in that direction to identify eligible women for interview. The interviewers then returned to the central point and repeated the aforementioned process until the required number of interviews had been obtained. In total, 6,785 women—2,161 in Bogra, 2,499 in Faridpur, and 2,125 in Moulvibazar—were interviewed across the study area. The field supervisors cross-checked 10% of the questionnaire, and data were double-entered to minimize errors at the inputting stage.

Data were collected as part of a baseline survey for a factorial cluster-randomized controlled trial to examine the effects of two interventions on newborn mortality. The first is a community-based participatory intervention with women's groups (10), and the second is an evaluation of the impact of training traditional birth attendants (TBAs) to prevent and manage birth asphyxia at the community level.

## RESULTS

### Background characteristics

Table 1 presents the background characteristics of the women interviewed. The majority (63.5%) of the women were in their twenties, 13.9% were aged less than 20 years, 21.3% were in their thirties, and 1.3% were aged over 39 years. Moulvibazar and Faridpur had a similar age distribution, however, Bogra had a higher percentage of interviewees aged less than 20 years. 85.5% of the interviewees were Muslims; this percentage was lowest in Moulvibazar, where 66.3% were Muslims and 33.6% were Hindus. 48.3% of the interviewees had no education, 29.8% had attended primary school, and 21.9% had attended secondary school, or higher. Only 18.3% of women in Moulvibazar attended secondary school or higher. Most (94.9%) interviewees were housewives and did not undertake any paid labour, and the percentage employed was highest (9.9%) in Moulvibazar.

**Table 1.** Background characteristics of women interviewed

Characteristics	Total (n=6,785) (%)	Bogra (n=2,161) (%)	Faridpur (n=2,499) (%)	Moulvibazar (n=2,125) (%)
Age (years)				
<19	13.9	27.9	11.9	2.2
20–29	63.5	59.2	64.4	66.7
30–39	21.3	11.7	22.5	29.5
40+	1.3	1.2	1.2	1.6
Religion				
Muslim	85.5	91.7	96.4	66.3
Hindu	14.5	8.3	3.6	33.6
Christian	0.0	0.0	0.0	0.1
Education				
No education	48.3	48.6	49.2	47.1
Primary education (complete + incomplete)	29.8	28.5	26.7	34.6
Secondary education +	21.9	22.9	24.1	18.3
Employment status				
Housewife	94.9	99.1	95.5	89.9
Employed	5.0	0.9	4.4	9.9
Student	0.1	0.0	0.1	0.2

### Antenatal care

Table 2 shows the total number of antenatal care check-ups attended and the timing of the first check-up by trimester of pregnancy. Forty-seven percent of the study women received at least one antenatal care check-up during their pregnancy, 19% received the Bangladesh's nationally-recommended antenatal care check-ups (at least three), and 11% received the World Health Organization-recommended antenatal care check-ups (four or more). Some district-level variation existed, mainly between Bogra and the other two districts. Bogra had the lowest percentage (36%) of women attending at least one antenatal care check-up and the lowest percentage (6%) attending four or more. The reasons given for never attending an antenatal check-up included: no perceived need, not knowing where to go, afraid to go, no-one to accompany, family forbade, too busy, too far away, too expensive, attitude of providers, lack of trust of providers, no provider available, and preferring visit to kabiraj (traditional healer) instead.

**Table 2.** Uptake of antenatal care

Antenatal care visit	Total (n=6,785) (%)	Bogra (n=2,161) (%)	Faridpur (n=2,499) (%)	Moulvibazar (n=2,125) (%)
Total number of antenatal care visits				
None	53.0	64.4	50.5	44.2
1	14.6	13.8	14.4	15.5
2	13.3	9.5	10.8	20.2
3	8.3	6.2	8.9	9.8
4+	10.8	6.1	15.4	10.3
Timing (months) of first antenatal care visit	(n=3,245)	(n=781)	(n=1,266)	(n=1,198)
1–3	24.9	19.9	25.4	27.7
4–6	47.8	42.1	49.6	49.5
7+	27.3	38.0	25.0	22.8

Overall, 25% of those who attended antenatal care check-ups had their first check-up in the first trimester of pregnancy, 48% in the second trimester, and 27% waited until the last trimester of pregnancy. In Bogra, 20% had their first visit in the first trimester and 38% waited until the third trimester before accessing antenatal care for the first time.

Women were asked about their uptake of tetanus toxoid (TT) before and during pregnancy. Table 3 presents a classification, which differentiates among those who have no protection against tetanus, those who are partially protected, and those who are fully protected. The classification divides women into four groups: those who have never had a TT injection; those who did not have one during their last pregnancy and although they had previously had at least one injection, they have not had the full five-dose lifetime protection; those who had at least one during their last pregnancy but again had not had the five-dose lifetime protection; and finally, those who were considered fully protected by either having two during their last pregnancy or at least five prior to their pregnancy. The results are encouraging as 69% of the women could be considered to be fully protected, and a further 18% had at least one TT injection during their last pregnancy across the study area. In contrast to other variables presented in this paper, the uptake in Bogra was highest with 75% fully protected, and a further 15% had one injection during the last pregnancy. A greater difference was observed in Moulvibazar, where 60% could be considered to be fully protected, while 19% did not have any injections during their last pregnancy and were not considered to have lifetime protection. The reasons women gave why they had never had a TT injection included: no perceived need, too scared, not given by health centre/provider, unaware of the necessity, forbidden by family members, fear of miscarriage, too busy, live too far away, and too shy.

**Table 3.** Uptake of tetanus toxoid immunization and consumption of iron tablets during pregnancy

Uptake	Total (n=6,785) (%)	Bogra (n=2,161) (%)	Faridpur (n=2,499) (%)	Moulvibazar (n=2,125) (%)
Uptake of tetanus toxoid				
Never had tetanus toxoid	6.9	4.8	5.6	10.5
No tetanus toxoid in last pregnancy and 1–4 previously	6.5	5.1	5.8	8.8
1 in last pregnancy and 0–4 previously	17.8	15.0	17.4	21.1
2 in last pregnancy or ≥5 previously	68.8	75.1	71.2	59.6
Length **(months) of time consumed iron tablets**				
Never took iron tablets	48.4	61.4	41.7	42.9
<1	17.2	22.3	17.5	11.8
1–3	16.0	9.7	19.8	17.8
4–6	11.1	3.6	14.6	14.4
>6	6.8	2.9	5.4	12.2

Women were asked about whether they had taken any iron tablets during their pregnancy and, if so, for how long (Table 3). Fifty-two percent of the study women took iron tablets during pregnancy. Women in Bogra had the lowest (39%) iron-tablet consumption and the lowest percentage (16%) consuming iron tablets for more than one month. Side-effects that the women experienced as a result of consuming iron tablets included hyperacidity, nausea/vomiting, constipation and foul-smelling black stools.

### Delivery

Table 4 shows that over 90% of births in the study area were delivered at home. Of those which were delivered in a facility, most (7%) took place in the government facilities, while only 2% occurred in private facilities and 0.5% in non-government organization (NGO) facilities. The pattern was similar across the districts, although the highest percentage (93%) of deliveries at home was observed in Bogra, and the highest percentage (10%) of deliveries occurred in the government facilities in Faridpur.

**Table 4.** Place of birth and attendance at delivery

Place of and attendance at delivery	Total (n=6,785) (%)	Bogra (n=2,161) (%)	Faridpur (n=2,499) (%)	Moulvibazar (n=2,125) (%)
Place of delivery				
Home	90.4	93.4	87.1	91.4
Government	6.5	3.6	9.6	5.6
Private	2.1	1.6	2.2	2.5
NGO	0.5	1.2	0.4	0.0
Other	0.5	0.2	0.7	0.5
Delivery attendant				
Doctor/nurse	11.1	6.5	15.8	10.2
Other health workers*	1.0	0.5	1.6	0.6
TBA	52.7	49.1	43	67.8
Friend/relative	34.4	43.6	38.6	20.1
Others	0.8	0.3	1.0	1.3

*Grouped together, which included Sub-Assistant Community Medical Officers, Family Welfare Visitors, Family Welfare Assistants, Female Health Assistants, paramedics, and outreach workers, and were government, private, and NGO employees

Table 4 also shows the distribution of births by delivery attendant. Deliveries by doctors or nurses across the study area were low (11%), particularly in Bogra (6.5%). All other health workers were grouped together, which included Sub-Assistant Community Medical Officers (SACMOs), Family Welfare Visitors (FWVs), Family Welfare Assistants (FWAs), Female Health Assistants (FHAs), paramedics, and outreach workers, and were government, private, and NGO employees. Overall, they assisted in 1% of the deliveries, with little variation between the districts. Fifty-three percent of births were delivered by traditional birth attendants (TBAs), and 34% were delivered by friends or relatives. Moulvibazar had the highest percentage (68%) delivered by TBAs and the smallest percentage (20%) delivered by friends or relatives.

Due to the large number of births at home, it is important to assess whether hygienic practices are being implemented in the community. The mothers were, thus, asked whether the birth attendant washed their hands with soap prior to delivery, whether the instrument used for cutting the umbilical cord, and whether the thread used for tying the cord were boiled prior to use. Eighteen respondents were not aware whether these hygienic practices had taken place or not; Table 5 presents the results for the remaining women.

**Table 5.** Hygiene practices at delivery

Practice	Total (n=6,116) (%)	Bogra (n=2,008) (%)	Faridpur (n=2,173) (%)	Moulvibazar (n=1,935) (%)
Washed hands with soap before delivery	66.5	48.0	63.0	89.6
Boiled instrument used for cutting umbilical cord	61.4	33.2	64.0	87.9
Boiled thread used for tying umbilical cord	57.1	30.5	59.0	82.6
All of the above	53.8	27.6	53.3	81.6
None of the above	29.4	47.6	30.1	9.7

All the three hygienic practices were implemented in 54% of deliveries in the overall study area compared to none of the practices implemented in just under a third of deliveries. There was little variation among which hygienic practices were undertaken, with 67% washing their hands with soap before delivery, 61% boiling the instrument, and 57% boiling the thread. There were, however, variations in districts in the uptake of hygienic practices. Moulvibazar showed 82% undertaking all the three practices, whereas, in Bogra, the uptake of hygienic practices in deliveries at home was very low. None of these hygienic practices was implemented in 48% of deliveries in Bogra, and all the three hygienic practices were implemented in only 28% of deliveries. The boiling of blade and thread was low in Bogra, with only 33% and 31% practising this respectively.

### Postnatal thermal care and breastfeeding

Overall, 59% of the infants were wiped, and 64% were wrapped immediately after delivery but there was a considerable variation in districts (Table 6). Bogra had the highest percentage of infants being wiped (73%) and wrapped (76%) immediately after birth, while Moulvibazar had by far the lowest (43% and 45% respectively).

**Table 6.** Thermal care of newborn infants

Thermal care	Total (n=6,785) (%)	Bogra (n=2,161) (%)	Faridpur (n=2,499) (%)	Moulvibazar (n=2,125) (%)
Infant was wiped immediately	58.9	72.5	59.0	45.1
Infant was wrapped immediately	64.3	75.9	72.0	43.4
Timing (hours) of first bathing of infant				
Immediately	43.8	31.6	46.6	53.1
<6	6.9	8.0	4.1	9.0
7–24	14.7	24.7	7.8	12.6
>24	30.6	31.6	38.1	20.7
Don't know	4.0	4.1	3.4	4.6

Table 6 presents the results of when infants were bathed for the first time. Ideally, infants should not be bathed until at least 24 hours after delivery to maintain body temperature and minimize the risk of hypothermia, but across the study area, 65% of the infants were bathed before this time limit, and 44% were bathed immediately after birth. Moulvibazar had the highest percentage of the infants bathed immediately after birth, with 53% of the infants were bathed within 24 hours, while Bogra had the lowest percentage (32%). In all the districts, there appears to be two patterns with regard to the bathing of infants, in that they are either bathed immediately (44%) or not until after 24 hours (31%).

Women were asked about whether their infants were ever breastfed, whether colostrum was their first food, and what the timing of first breastfeeding was (Table 7). In terms of ever-breastfeeding, the results were impressive, with an average of 96% in the overall study area and a very little variation in districts. The results for being fed colostrum as a first food were less promising, with 43% overall and only 27% in Bogra. Most infants who were not fed colostrum as their first food were given honey, sugar water, or plain water. Twenty-eight percent of the infants were put to the breast immediately, with 48% within the first hour, while 22% waited for more than 24 hours after delivery. There was some degree of variation in districts: Faridpur performed the best, and Bogra faired worst, with nearly half of the mothers not putting the baby to the breast until after 24 hours.

**Table 7.** Breastfeeding practices

Practice	Total (n=6,785) (%)	Bogra (n=2,161) (%)	Faridpur (n=2,499) (%)	Moulvibazar (n=2,125) (%)
Ever breastfed infant	96.3	97.1	97.6	95.9
Infant fed colostrums as first food	42.2	26.5	54.8	43.4
Timing of first breastfeeding of infant				
Immediately after birth	28.3	20.0	39.8	23.2
<1 hour after birth	19.4	6.2	15.7	37.4
Between 2^nd^ and 6^th^ hour after birth	13.2	12.2	11.6	16.1
Between 7^th^ and 24^th^ hour after birth	3.9	4.9	4.6	2.0
Between 2^nd^ and 3^rd^ day	16.6	36.6	9.1	5.0
After 3^rd^ day	5.7	9.1	4.3	3.8

### Postnatal care

Table 8 shows the percentage of infants seen by a health provider in the first 24 hours. Overall, a provider saw 39.3% of the infants. There was a slight variation in districts, with Bogra having the highest percentage (44.2%) and Moulvibazar having the lowest percentage (31.5%). 10.4% were seen either by a doctor or by a nurse, 10.1% by another health worker, 16.2% by a TBA, and 2.6% by another provider. The latter included village doctor, homeopath, *kabiraj*, and spiritual healer. There was a lot of variation among districts as infants in Bogra were most likely to be seen by a TBA (31.1.%), infants in Faridpur were seen either by other health workers (25.5%) or by a doctor or by a nurse (13.5%), and infants in Moulvibazar were seen either by a TBA (18%) or by a doctor or by a nurse (10.6%).

**Table 8.** Postnatal check-up of infants

Postnatal check-up	Total (n=6,785) (%)	Bogra (n=2,161) (%)	Faridpur (n=2,499) (%)	Moulvibazar (n=2,125) (%)
Infant seen by a health provider in first 24 hours	39.3	44.2	41.6	31.5
Who saw the infant?				
Doctor/nurse	10.4	6.6	13.5	10.6
Other health workers	10.1	0.9	25.5	1.2
Traditional birth attendant	16.2	31.1	1.8	18.0
Others	2.6	5.6	0.8	1.7

## DISCUSSION

The results of this study indicate that, despite some degree of variation in districts, maternal and newborn-care practices during delivery remain a cause of concern in rural Bangladesh. The results come from a retrospective, cross-sectional baseline survey. Despite limiting the interviewees to those who had delivered in the previous year, recall-bias may have affected the accuracy of the results (11). The project is presently collecting prospective data in the same study area, which will enable comparison between the two methods of data collection, although any comparison will need to take into account changes over time.

The uptake of antenatal care was very low: 53% of the women interviewed did not have any antenatal care check-ups, while most of those who attended waited until late in their pregnancy and did not have the national or international recommended number of visits. Furthermore, the use of these indicators provides no information about the quality of care received by those who attended. The coverage of TT was good, most women either had at least one injection during their last pregnancy or had already completed their lifetime protection dosage. It is difficult to measure how protected a woman and her baby are against tetanus without taking into account the timing of the injections and the total number received. Despite the government policy that all women should take iron tablets as soon as pregnancy is detected and continue throughout pregnancy, only half of our interviewees took any iron tablets and only 18% for longer than four months. Some women experienced problems in obtaining either their TT shot or iron tablets either from a health facility or from a provider, suggesting a need to address the supply side. Others did not perceive a need, suggesting that improved health education may help women to understand the benefits and side-effects, such as digestion problems, after taking iron tablets.

The number of deliveries attended by doctors or nurses was small (11%). All other government, NGO and private health professionals were grouped together as the percentage attended by any of these was negligible. Furthermore, when the women were asked to distinguish between different health workers, they might have been unsure of their exact profession, and the breakdown might have been inaccurate. Likewise, TBAs were not categorized into trained and untrained as there is no clear national definition regarding these classifications. A clear definition would need to consider factors, such as content of training, length of training, ability at the end of training, and time since training. Even if a clear definition was in place, women in the community may not know the exact details of the training background of the TBAs, and if they did, whether that would classify them as trained or not.

This study further supports findings of other studies that the overwhelming majority of deliveries in rural Bangladesh take place at home and are not attended by health professionals. The Bangladesh Maternal Health Services and Maternal Mortality Survey (BMMS) showed similar percentages of women in rural areas being delivered in facilities and by health professionals (3). A bigger difference was seen with regard to whether a TBA, or a friend, or a relative delivered the baby. This survey showed that 34% of the women were delivered either by a friend or by a relative compared to 12% in the BMMS (3). This may be a valid difference based on the areas included in this study being different to the national rural average or it may again be due to problem of definition, i.e. when does a woman who delivers an infant for her relatives to become a TBA. Despite any confusion the problem of definition may cause, there is no doubt that most infants are not being delivered by skilled birth attendants (SBAs) in an enabling environment.

Currently, there is a considerable debate that resources should be targeted towards training SBAs and that there is little merit in training TBAs (12–14). The Government of Bangladesh stopped training TBAs in July 1998 and decided to introduce SBAs in the community, which is one of the key interventions for human resource development under the Bangladesh Maternal Health Strategy 2000. They started training FWAs and FHAs to become SBAs in mid-2003, after this survey was conducted, and, by November 2005, had trained 1,276 FWAs and FHAs from 24 districts. There is currently concern from the evaluation of the pilot, and to achieve their goal of having 50% of deliveries attended by skilled attendants by 2010 (15), the Government needs to train up to 25,000 more SBAs. This target is ambitious and, at the current rate, it could take 30 years or more before there is a full coverage of SBAs across the country. There is a lack of rigorous evidence to support the opinion regarding the discontinuation of TBA training (16, 17) and randomized controlled trials with enough power to look at the impact of TBA training on neonatal and maternal mortality would help address this issue.

A study in Bangladesh has shown that, although rates of infection did not decrease, training TBAs does make a difference to their behaviour, with 45% of trained TBAs practising the three cleanings (hand-washing with soap, clean cord care, clean surface) compared to 19% of untrained TBAs (18). This change in behaviour is supported by a meta-analysis, which also found a reduction in peri-neonatal mortality in births attended by trained TBAs compared to untrained TBAs (19). Recently, results of a randomized controlled trial in Pakistan showed that the training of TBAs and their integration within an improved health system were effective in reducing perinatal mortality by 30% and maternal mortality by 26%, although the latter did not achieve any statistical significance (20).

Our finding that over one-third of the infants were taken for a postnatal check-up within 24 hours of birth is more encouraging. This suggests an increasing awareness of the need to seek help for neonatal problems and might be a contributory factor to the declining rate of neonatal mortality in Bangladesh despite the low coverage of skilled birth attendance.

More attention needs to be focused on how countries such as Bangladesh have succeeded in reducing the rate of maternal mortality with such a low percentage of deliveries attended by skilled attendants. In addition to a long-term goal towards skilled attendance at delivery, interim strategies should be considered in the community given the high numbers of deliveries occurring in the community, and the fact that the coverage of SBAs is very low and is likely to remain so for the foreseeable future. The community strategies could also increase the number of infants receiving essential newborn care, being wiped, wrapped, and fed colostrum immediately after delivery, while decreasing the number that are bathed in the first 24 hours and increasing the number exclusively breastfed for the first six months.

In conclusion, the findings of our study support the need for more targeted approaches to changing maternal and newborn-care practices home, if further reductions in maternal and newborn mortality are to be achieved and Bangladesh is to make progress towards Millennium Development Goals 4 and 5.

## ACKNOWLEDGEMENTS

The Department for International Development of the United Kingdom and the UK Community Fund funded this study. The funders can accept no responsibility for any information provided or views expressed in this paper. The authors would like to thank many individuals in Bogra, Faridpur, and Moulvibazar who gave their time generously and without complaint, and all of the field staff of the DAB Perinatal Care Project (PCP), namely the three District Managers—Golam Azam, Rezaul Alam Chowdhury, and Mubinul Karim—without whom this study would have been impossible. This paper is dedicated to the memory of Dr. Mohammed Kamruzzaman, the PCP Project Manager at the time of the baseline survey, who died in 2003. The authors declare no conflict of interest.
